# The case for increasing diversity in tissue-based functional genomics datasets to understand human disease susceptibility

**DOI:** 10.1038/s41467-022-30650-8

**Published:** 2022-05-25

**Authors:** Erping Long, Montserrat García-Closas, Stephen J. Chanock, M. Constanza Camargo, Nicholas E. Banovich, Jiyeon Choi

**Affiliations:** 1 Division of Cancer Epidemiology and Genetics, National Cancer Institute, National Institutes of Health, Bethesda, MD USA; 2 The Translational Genomics Research Institute, Phoenix, AZ USA

**Keywords:** Gene expression profiling, Genetic variation

## Abstract

Tissue-based functional genomics resources including molecular quantitative trait loci datasets lack diversity in ancestry and tissue types and thus are inadequate for comprehensively investigating gene regulation. Global efforts to increase the tissue diversity will help achieve more equitable medical care.

Investigating the molecular basis of disease susceptibility is a central question in human genetics, which, in turn, should inform strategies for precision medicine and prevention. While genome-wide association studies (GWAS) have identified thousands of disease/trait-associated loci, the mechanistic underpinnings have been adequately explored in a small fraction^1^. Since most GWAS loci function through alterations of gene regulation, usually in a cell-type and context-specific manner, tissue-based functional genomics studies hold great promise to inform the functional consequences. To this end, expression quantitative trait loci (eQTL) and other QTLs of molecular phenotypes (e.g., DNA methylation, alternative splicing, and chromatin features) have been widely used to investigate the functional bases of GWAS loci. To date, the ability to use molecular QTLs to evaluate GWAS loci has major limitations due to the limited sample size and the lack of diversity in tissue-based studies. This lack of diversity is a major reason that we are currently unable to fully characterize the effects of GWAS variants.

## Samples from diverse ancestries and tissue types are needed

**Fig. 1:**
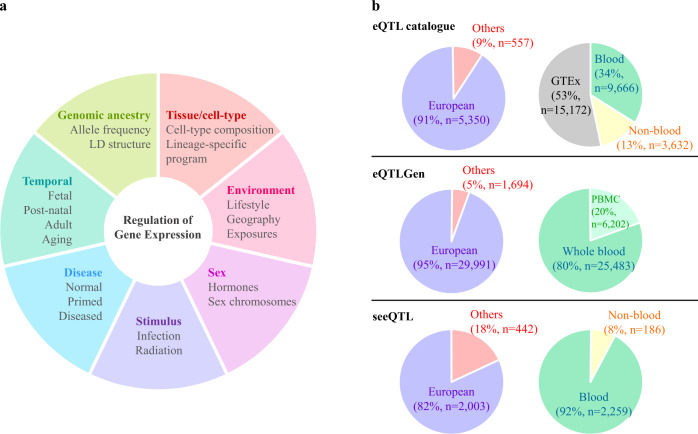
Factors influencing genetic regulation of gene expression and a current snapshot of eQTL resources. **a** Diverse factors and contexts influencing genetic regulation of gene expression are highlighted. **b** Current statistics of ancestry and tissue/cell-type information from three representative eQTL dataset aggregation resources are summarized. Europeans in eQTLGen include studies of predominantly Europeans. Links to the resources are listed in Box 1.

The Genotype-Tissue Expression (GTEx) project has pioneered efforts to catalog genetic variants and molecular phenotypes of human tissues by collecting normal (non-diseased) tissue samples representing different organs from many donors, mainly of European ancestry. From their findings, together with other studies, the dynamic and highly context-specific nature of gene expression regulation in human tissues has emerged as a major theme, one that is critical to pursue to understand the functional underpinnings of GWAS loci (Fig. 1a). Specifically, eQTL studies based on purified/cultured cells, computational cell-type deconvolution, or single-cell sequencing identified cell-type-specific and cell-type-interacting molecular QTLs^2^. Notably, eQTLs were reported to vary across populations, which could be attributed to genetic factors (e.g., differences in allele frequency and linkage disequilibrium) as well as distinct lifestyle and environmental exposures. Genetic regulation of gene expression could also be influenced by sex, temporal factors, and stimuli. Because multiple factors contribute to gene expression regulation, it is imperative to obtain tissues representing the breadth of these diverse contexts to have a comprehensive understanding of disease susceptibility. In this regard, differences in ancestral background and tissue/cell types, and environmental exposures should be sought. Further, the analyses need to be performed in sufficiently large sample sizes to explore the underlying biology and draw statistically robust conclusions.

## Tissue-based functional genomics resources lack diversity

The current tissue-based datasets lack diversity in a few critical aspects. Most of these resources are from individuals of European ancestry. In the latest version of the GTEx project (v8), 85% of 948 donors are of European descent. A recent aggregation of the major eQTL studies by eQTL catalogue showed that 91% of 5907 individuals from 19 studies are of European descent, and only 6 studies also included individuals of other ancestries^3^. Similar European-centric collections dominate most consortium-based efforts such as eQTLGen (34 of 37 studies and 95% of 31,685 individuals are predominantly of European ancestry^4^) or datasets compiled in seeQTL browser (82% of 2445 samples are of European descent^5^; Fig. 1b). There could be complex underlying reasons for this disparity, a subset of which we attempt to describe here. Potentially one of the most important factors is that large-scale genetic cohorts where tissue resources tend to come from are typically built upon infrastructure favoring individuals of European ancestry. This could also be driven by funding disparities^6^ mainly affecting researchers of underrepresented populations, lack of trust in scientific research^7^ by potential donors from underrepresented populations, and limited efforts to include research centers/hospitals that mainly serve underrepresented populations. All of these are also closely tied with structural racism. These issues can be exacerbated in low and middle-income countries (LMIC). To generate high-quality mRNA for eQTL analysis rigorous inclusion criteria and standardized procedures are required throughout the tissue collection and preparation process, which are more challenging in LMIC with limited infrastructure and professional training.

Because of the current European-centric tissue collection, GWAS of non-European or admixed populations often face analytical challenges in QTL integration, as most tools including co-localization and transcriptome-wide association study (TWAS) assume matching and homogeneous ancestry. In an ancestry-stratified eQTL analysis of African, Hispanic/Latino, and European populations, expression prediction performance differed across populations for a subset of genes^8^. In another study, African- and European-ancestry-specific prediction models of breast tumor samples performed poorly across the ancestry but led to African ancestry-specific TWAS findings^9^. Further, the analyses of samples from non-European individuals in GTEx dataset identified population-biased eQTLs^10^ and influence of ancestry on colocalization^11^ for a small subset of variants/genes.

Another major issue is the lack of diverse cell types and biological contexts represented in population-scale datasets. While the tissue resources generated by GTEx and others have proved a valuable tool, these bulk tissue approaches obscure the cell-type heterogeneity within the organ. Indeed, simultaneous profiling of individual cell types in tissue samples provides us opportunities to study diverse biological contexts interacting with germline variation. The Human Cell Atlas (HCA) is leading an effort to create a reference map of all cell types in the healthy human body^12^, which currently established 18 organ-specific biological networks, including special initiatives towards developmental cell types, organoids, and overall genetic diversity. Despite these efforts, existing single-cell-based datasets of normal human tissues (e.g., Single Cell Expression Atlas^13^) still lack representation in geographical locations, genetic ancestries, and environmental exposures (e.g., smoking) that could contribute to gene expression and cell type variabilities. Furthermore, both single-cell and bulk datasets amenable to QTL analyses, due to sample size requirements, are heavily skewed towards blood-derived sample types or cultured cells (Fig. 1b). Indeed, the newly launched single-cell eQTL consortium (sc-eQTLGen)^14^, while an important first step, is focused on blood-derived cell types, and extension to other tissue types is crucial. This lack of representation of diverse cell types and contexts can be attributed to difficulties in medical accessibility to certain age groups and organ types as well as technical difficulties in obtaining hard-to-dissociate and more vulnerable tissue types during tissue processing. Further, epidemiological data informing the type and level of major exposures or infections associated with tissue specimens are logistically challenging to collect. While it should be noted that this disparity is further exacerbated in non-European populations and presents additional difficulties when working in LMIC, commitment and resources should be directed to develop and maintain state-of-the-art tissue-based collections.

## Toward increasing diversity in tissue resources

In line with the ongoing international efforts to address the current disparities, we suggest the following key approaches to increase diversity in tissue resources for functional genomics studies.

First, focused efforts should be made to collect and catalog samples from underrepresented populations and rarer tissue types by funding new initiatives, supporting and supplementing existing cohorts, and promoting international partnerships. For example, Chan Zuckerberg Initiative recently announced 33 projects to support single-cell atlas efforts through HCA by promoting the inclusion of diverse ancestries and pediatric tissue samples. A pilot initiative by the National Cancer Institute, the Multi-ancestry Cancer Genomics Atlas, is centralizing an individually small number of samples of underrepresented United States populations from multiple existing biobanks to achieve a sample size suitable for eQTL and other molecular studies. Further, creating and supporting tissue collection programs for the established ethnic minority cohorts (e.g., The Multiethnic Cohort Study^15^) could help establish organ-specific tissue datasets. Besides, international partnerships are crucial for empowering the underrepresented regions and populations to contribute to building diverse tissue resources. King Hussein Cancer Center has established the first ISO-compliant cancer tissue biobank in Jordan, benefiting from collaboration with Ireland and Switzerland^16^. Knowledge transfer and protocol standardization were also exemplified in The United States-Latin America Cancer Research Network^17^. In establishing such a partnership, it is important to put conscious efforts into building trust with the local communities in the LMIC through informed consent and benefit-sharing with the donors as well as training opportunities and equitable academic credit for the scientists. Funding and recognition of equitable academic partnership should be a high priority in pursuing these goals in a concerted effort to achieve global health equity in tissue-based functional genomics.

Second, technical and analytical development should accompany the sample collection efforts to maximize the benefit of accessible tissue resources. Technological advances allowing the use of flash-frozen or temperature-stable biobank samples in more diverse applications will help overcome the challenge of obtaining fresh tissues from new sources and benefit LMIC with limited infrastructure for cryopreservation. For example, single-nucleus preparation^18^, sample fixation, and tissue microdissection techniques^19^ from frozen tissues are expanding the range of cell-type-specific applications for using archival samples. Further, creating virtual tissue cohorts by addressing technical challenges in data integration across multiple tissue datasets could help boost statistical power for rarer tissue specimens. Furthermore, the development of more versatile statistical approaches allowing the use of molecular QTL and GWAS datasets from multi-ancestry and admixed populations will be invaluable in integrating the data collected from diverse ancestral populations.

Collective international efforts to reduce disparities in tissue resources involving patients, clinicians, and researchers will help us ultimately achieve more equitable medical care informed by well-balanced genetic research.

Box 1 Resources and initiatives mentioned in this comment

- **QTL resources**
  eQTL Catalogue, https://www.ebi.ac.uk/eqtl/
  eQTLGen, https://eqtlgen.org/
  seeQTL, https://www.seeqtl.org/
- **Single-cell resources**
  Human Cell Atlas, https://www.humancellatlas.org/
  Single-cell Expression Atlas, https://www.ebi.ac.uk/gxa/sc/home
  Single-cell eQTL consortium, https://eqtlgen.org/sc/
- **Mentioned initiatives**
  Chan Zuckerberg Initiative, https://chanzuckerberg.com/
  The Multiethnic Cohort Study, https://www.uhcancercenter.org/mec
  The United States-Latin America Cancer Research Network, https://www.cancer.gov/about-nci/organization/cgh/blog/2014/uslacrn-clinical-cancer-research
